# *Plaat1* deficiency reduces cardiac cardiolipin content and impairs exercise tolerance

**DOI:** 10.1016/j.jlr.2025.100822

**Published:** 2025-05-08

**Authors:** Ashkan Hashemi, Ming Rong Liu, John Z. Chan, Antonia N. Berdeklis, Alex D. Cocco, Michelle V. Tomczewski, Douglas Strathdee, Ken D. Stark, Robin E. Duncan

**Affiliations:** 1 University of Waterloo, Faculty of Health, Department of Kinesiology and Health Sciences, Waterloo, ON, Canada; 2 Transgenic Technology Laboratory, Cancer Research UK Beatson Institute, Glasgow, Scotland, UK

**Keywords:** cardiolipin, cell signaling, glycerophospholipids, phospholipids/metabolism, heart, knockout mice, lipids, mitochondria, oxidative capacity, phospholipase A and acyltransferase 1 (PLAAT1)

## Abstract

Phospholipase A and acyltransferase 1 (PLAAT1) catalyzes O-transacylase, N-transacylase, and phospholipase A_1/2_ reactions. We have demonstrated that PLAAT1 has O-transacylase activity in vitro using phosphatidylcholine as an acyl donor and monolysocardiolipin (MLCL) as an acyl acceptor, generating cardiolipin. However, a role for PLAAT1 in cardiolipin regulation in vivo has not yet been reported. We generated *Plaat1*-deficient (*Plaat1*^*−/−*^) mice and studied males and females for gross morphological differences, food intakes, respiratory gas exchange, total energy expenditure, and voluntary activity. We also evaluated cardiac cardiolipin contents, levels of mitochondrial proteins, and exercise capacity. Sex-matched *Plaat1*^*−/−*^ mice had highly similar body weights to their wild-type (*Wt*) littermates, although male *Plaat1*^*−/−*^ mice ate less. Male and female *Plaat1*^*−/−*^ hearts were 14.2% and 10.6% smaller, respectively. Cardiac cardiolipin levels were ∼one-third lower in male and female *Plaat1*^*−/−*^ mice compared to their sex-matched *Wt* littermates, largely due to lower cardiolipin linoleate. Levels of the mitochondrial protein succinate dehydrogenase complex flavoprotein subunit A were 13.8% and 16.3% lower in male and female *Plaat1*^*−/−*^ mice, respectively. Both male and female *Plaat1*^*−/−*^ mice had significantly lower oxygen consumption, carbon dioxide production, and total energy expenditure, and male *Plaat1*^*−/−*^ mice had lower rearing activity than their sex-matched *Wt* littermates. While other measures of voluntary activity, including locomotion and ambulation did not differ significantly between genotypes, both males and females had reduced exercise tolerance. This work demonstrates a critical role for PLAAT1 in cardiac cardiolipin content and the regulation of energy metabolism and exercise tolerance in vivo.

Phospholipase A and acyltransferase (PLAAT) 1 is a member of the PLAAT family of five enzymes identified in humans (PLAAT1-5), with three also conserved in rodents (PLAAT 1, 3 and 5) (1). *Plaat1* tissue expression profiles are broadly similar across species surveyed, with highest levels found in heart, skeletal muscle, brain, and testes (2). All PLAAT enzymes, including PLAAT1, have been found to catalyze multiple types of phospholipid metabolism reactions in vitro.

In studies using purified enzyme, Shinohara and colleagues have identified in vitro phosphatidylcholine (PC):lysophosphatidylcholine (LPC) O-transacylase activity, phospholipase A_1/2_ (PLA_1/2_) activity using PC as a substrate, and *N*-transacylase activity using phosphatidylethanolamine (PE) as an acyl acceptor and PC as an acyl donor (3). In more recent studies, our group has also found that PLAAT1 has O-transacylase activity in vitro using PC as an acyl donor and monolysocardiolipin (MLCL) as an acyl acceptor, and that overexpression of this enzyme in cultured cells increases cardiolipin concentrations (2). However, the effect of loss of *Plaat1* on cell or tissue cardiolipin has not yet been reported.

To determine whether PLAAT1 deficiency alters tissue cardiolipin content and/or composition, we generated a *Plaat1* knockout (*Plaat1*^*−/−*^) mouse model and evaluated the cardiac cardiolipin profile. As an integral component of the inner mitochondrial membrane, cardiolipin plays an important role in the formation of cristae and stabilization of electron transport chain components needed for energy metabolism (4, 5), which in turn influences growth (6), health (7, 8), lifespan (6), cardio- and respiratory metabolism (9), and exercise capacity (6, 10), among other measures. Thus, in addition to evaluating the cardiac cardiolipin profile of this model, we also performed a series of studies to evaluate morphological, metabolic, and physiological characteristics. Here we report that deficiency of *Plaat1* profoundly decreases total heart cardiolipin content in both male and female mice and also decreases exercise tolerance and alters metabolic measures. Our findings are discussed in the context of the known functions of this enzyme as well as in light of recent work on other animal models of *Plaat1* deficiency.

## Materials and Methods

### Animals

*Plaat1*^*−/−*^ mice lacking exon 3 were generated in C57Bl/6J zygotes using CRISPR and are described in Appendix 1, Fig. S1, and confirmed by direct sequencing. Exon 3 was targeted because deletion of this region will disrupt enzymatic activity of the PLAAT1 protein, since a catalytically critical cysteine residue is present in this region (1), as illustrated in Fig. S1. Mice were housed at the central animal facility at the University of Waterloo, provided with standard chow and water ad libitum, and maintained in a 12 h light-dark cycle. At 8 weeks of age, mice underwent assessment by indirect calorimetry, and at 12 weeks of age, mice performed the treadmill exercise capacity test. From 19-20 weeks of age, food intake was measured daily for 7 days. At 20–30 weeks of age, a subset of littermate wildtype (*Wt*) and *Plaat1*^*−/−*^ mice were sacrificed, and organs and tissues were excised, weighed, and snap-frozen in liquid nitrogen and then stored at −80°C. *Tafazzin*-deficient mice (*Tafazzin*^*−/Δ*^) have previously been described (6). Animal procedures with *Plaat1*^*−/−*^ mice (AUPP#43325, #43442) and *Tafazzin*^*−/Δ*^ mice (AUPP#41822, #43431) were performed with ethical approval from the University of Waterloo Animal Care Committee and comply with guidelines of the Canadian Council on Animal Care. The generation and maintenance of the *Tafazzin*^*−/Δ*^ mice was approved under a UK Home Office Licence (PP9886217).

### Lipid extraction, thin layer chromatography (TLC), and gas chromatography (GC)

Tissue lipid extraction, TLC, and GC were performed essentially as we have previously described (10). Mouse heart tissue was homogenized in phosphate-buffered saline (PBS) using a TissueLyser II (Qiagen Canada) for 2 min at a frequency of 25 Hz. Total lipids were extracted from the homogenate by the addition of 2:1 (v/v) chloroform: methanol with the antioxidant butylated hydroxy-toluene (BHT) overnight. The next day, PBS was added and the samples were vortexed and then centrifugated at 3000 *g* for 5 min to separate the organic and aqueous phases (10). The lower organic phase was extracted to a new tube. Samples were dried under a gentle nitrogen stream then reconstituted in 50 μl of chloroform and spotted on a silica gel HF plate (20 cm × 20 cm, 250 μM; Analtech Inc., Cole-Parmer Canada), and resolved using a chloroform:methanol:2-propanol:0.25% KCl: trimethylamine (30:9:25:6:18, v/v/v/v/v) solvent front. Cardiolipin bands were visualized by UV illumination after spraying with 0.1% 2,7-dichlorofluorescein in methanol (w/v) and identified and scraped based on comparison with a cardiolipin standard (Avanti Polar Lipids, Millipore Sigma).

In preparation for gas chromatography (GC), the fatty acyl species within cardiolipin were derivatized to fatty acyl methyl esters by a transesterification method using 14% boron trifluoride in methanol and hexane (Thermo Scientific), and nonadecanoic acid (19:0) ethyl ester was added as an internal standard (Nu-Check Prep). This mixture was heated at 95°C for 1 h and the product was centrifuged at 3000 *g* for 5 min, then the top layer was transferred to a fresh tube, dried under a N_2_ stream, and resuspended in 65 μl of heptane. Analysis using gas chromatography with flame ionization detection was performed using the Agilent 7890A gas chromatograph equipped with a DB-FFAP 15 m × 0.10 mm injected dose × 0.10 μm film thickness nitroterephthalic acid modified polyethylene glycol capillary column (J&W Scientific/Agilent Technologies) with hydrogen as the carrier gas. Briefly, 1 μl of sample was introduced by an Agilent 7693A autosampler into the injector and heated to a temperature of 250°C with a split ratio of 50:1. The initial temperature was 150°C with a 0.25 min hold followed by a 35°C/min ramp to 200°C, a 1 °C/min ramp to 211°C, and then an 80°C/min ramp up to 245°C with a 4-min hold at the end. The flame ionization detector temperature was set at 300°C with air and nitrogen make-up gas flow rates of 300 and 10 ml/min, respectively, sampled at a frequency of 50 Hz.

Fatty acyl composition was expressed both as concentrations (measured in μg of fatty acids per mg of tissue) and as relative mass percentages (wt/wt%) as a proportion of the total mass of fatty acids analyzed. Total cardiolipin concentration was calculated as the total mass of all cardiolipin fatty acyl species per mg of tissue analyzed.

### Western blot analysis

Western blot analysis was performed essentially as we have previously described with minor modifications (11). Briefly, tissues were prepared by homogenization using a TissueLyser II in RIPA buffer (50 mM Tris–HCl, pH 8.0; 150 mM NaCl, 1% Nonidet P-40, 0.5% sodium deoxycholate, and 0.1% SDS with 10 μl/ml of protease/phosphatase inhibitor cocktail (Cell Signaling). This mixture was incubated at 4°C for 30 min, then centrifuged at 10,000 *g* to pellet unbroken cells and debris. Protein concentrations in clarified lysates were determined using bicinchoninic acid solution. Samples were mixed with 6× Laemmeli Buffer (125 mM Tris–HCl, pH 6.8, 20% glycerol (v/v), 4% SDS (w/v), 10% 2-mercaptoethanol (v/v), and 0.05% bromophenol blue) and heated to 95°C for 5 min, then electrophoresed through 10% SDS-PAGE TGX Stain-Free™ Fast Cast™ gels (Bio-Rad Canada, Mississauga, Ontario, Canada) at 120 V for 1 h. Total loaded protein per lane was detected by fluorescence-activation of the TGX Stain-Free™ gel under UV illumination, imaged for relative density using a ChemiDOC Touch Imaging System (Bio-Rad Canada), and quantified using Image Lab software (Bio-Rad Canada). Proteins were then transferred onto nitrocellulose membranes using a Bio-Rad Trans-Blot Turbo system (Bio-Rad Canada) set at 25 V for 30 min. Membranes were blocked with 5% bovine serum albumin (BSA) in TBST (50 mM Tris–HCl, pH 7.4, 150 mM NaCl, 0.1% Tween-20) for 1 h at room temperature, then incubated overnight at 4°C in TBST containing 5% BSA and primary antibodies (1:1000 dilution) directed against heat shock protein 60 (HSP60), succinate dehydrogenase complex flavoprotein subunit A (SDHA), and cytochrome c oxidase subunit IV (COX IV) (Cell Signaling Technology). Membranes were then washed three times with TBST, incubated with horseradish peroxidase (HRP)-conjugated secondary antibodies (Cell Signaling Technology) diluted in TBST with 5% BSA (1:2000) for 1 h at room temperature, then washed again three times for 10 min per wash with TBST. Membranes were rinsed with 1 ml of LumiGLO (Cell Signaling Technology) for 1 min, followed by the detection of bands using a ChemiDOC Touch Imaging System (Bio-Rad Canada). Protein band densities were quantified using Image Lab software (Bio-Rad Canada) and normalized to total protein visualized per well.

### Real-time quantitative PCR

Total RNA was extracted from whole mouse hearts using TRIzol® Reagent (Invitrogen) according to the manufacturer’s protocol after tissue was mechanically disrupted using a POLYTRON® PT 1200 E homogenizer (VWR). RNA content was measured using a Nanodrop 2000 Spectrophotometer (ThermoFisher) and diluted so that 2 μg of RNA per 10 μl reaction was used for cDNA synthesis via random hexamer priming using a High-Capacity cDNA Reverse Transcription kit from Applied Biosystems according to the manufacturer’s protocol. cDNA (1 μl from a stock diluted 1:5) was mixed with PerfeCTa® SYBR® Green FastMix (Quanta Biosciences) and forward and reverse primers designed using mRNA reference sequences from the National Center for Biotechnology Information. A list of primers sequences is provided in Appendix 1 (Supplemental Table S3). Transcript levels were detected using a CFX-96 Connect Real Time qPCR System (BioRad) using thermal cycling conditions of 50°C for 2 min, then 95°C for 20 s, followed by 49 cycles at 95°C for 3 s, then 60°C for 30 s. Gene expression was analyzed using the ΔΔCt method, with the Ct values first normalized to a housekeeping gene (*ie* glyceraldehyde-3-phosphate dehydrogenase (*Gapdh*) for fibronectin (*Fn*) and *beta-actin* (*b-actin*) for all others) to determine the relative fold difference between *Wt* and *Plaat1*^−/−^ mouse hearts.

### Indirect calorimetry

The mice were subjected to indirect calorimetry using the Comprehensive Laboratory Animal Monitoring System (CLAMS, Columbus Instruments). Prior to CLAMS testing, mice were housed individually in standard cages for a duration of 24 h. Before initiating the CLAMS testing session, the gas sensors were adjusted using a standard gas mixture, comprising 20.5% oxygen, 0.5% carbon dioxide, with the balance made up of nitrogen. Gases were supplied to the chambers at a rate of 0.5 L/min, and the environment was maintained at 22–23°C, following a 12:12-h light/dark cycle (with lights on from 07:00 to 19:00 and off from 19:00 to 07:00). Throughout the investigation, the consumption of oxygen (VO_2_; ml/kg/h) and production of carbon dioxide (VCO_2_; ml/kg/h) within each chamber were measured at intervals of 28 min. The respiratory exchange ratio (RER) represents the ratio of VCO_2_ divided by VO_2_ and can be used to estimate the fuel source for energy production based on the difference in the number of oxygen molecules required for glucose versus fatty acid oxidation, where an RER of 0.7 indicates that fatty acids are the primary substrate, while an RER of 1.0 indicates that carbohydrates are the primary energy substrate (12, 13). The total energy expenditure (TEE) was calculated using the Lusk equation ((3.815 + 1.232 × RER) × VO_2_ (in liters)) (14), with the TEE values adjusted according to total body weight (kg). In addition to these measures, the chambers were fitted with photobeams situated above the cage floor, designed to track the total locomotor activity through infrared beam breaks along three planes: x (locomotion), y (ambulation), and z (rearing). Activity was measured by calculating the sum of movement counts. Assessments were carried out over a span of 26 h, where the initial 2 h period was allocated to allow the mice to adapt to the new equipment, and the data collected during this acclimatization phase were excluded from the 24 h and 12 h-light and 12-h dark cycle analyses.

### Treadmill exercise capacity and exercise recovery test

The exercise capacity of the mice was assessed through an incremental treadmill running test using a five-lane motor-driven treadmill (Model LE8700, Panlab/Harvard Apparatus, Barcelona, Spain), set at a constant incline of 5^◦^, as we have previously described (6, 10). Briefly, training and testing sessions were conducted during the evening hours (17:00–21:00) in a darkened room illuminated by overhead red lighting. The first three days were designated as an acclimatization period for the mice to become accustomed to the equipment. On the first and second days, the mice were positioned on the stationary treadmill for a period of 5 min, then gradually induced to run at incremental speeds of 5 cm/s for 5 min, 10 cm/s for 2 min, and then 15 cm/s for a duration of 3 min. On the third day, the mice were again placed on the static treadmill for 5 min, and then their training progressed with running speeds of 5 cm/s for 3 min, 10 cm/s for 2 min, 15 cm/s for 2 min, and concluding with 20 cm/s for a 3-min interval. The fourth day was assigned as a rest day. The exercise capacity test was conducted on day 5. Mice were initially set on the stationary treadmill for 5 min, and then the exercise capacity evaluation commenced at a starting speed of 10 cm/s. This speed was subsequently increased by 3 cm/s every 2 min, continuing to a maximum speed of 70 cm/s. The point of exhaustion was identified as an inability to continue running for a consecutive 5s period. Metrics including the total running time until reaching exhaustion, the average and maximum speed achieved, and the overall distance covered, were documented.

### Statistical analysis

Differences in the total content, tissue concentration, and relative mass% of fatty acyl species between male *Wt, Plaat1*^*−/−*^ and *Tafazzin*^*−/Δ*^ mice were analyzed using one-way ANOVA with Newman-Keuls post hoc test, while differences between female *Wt* and *Plaat1*^*−/−*^ mice were analyzed using Student’s *t* test. Differences between genotypes (within sexes) in immunodetectable mitochondrial proteins, normalized tissue weights, CLAMS measures, and exercise tolerance were analyzed by Student’s *t* test. Survival of mice is depicted using Kaplan–Meier survival distributions, and significance was analyzed by the log-rank Mantel-Cox test. When mice from either genotype reached 30 months of age, their data-point was entered as censored data.

## Results

### *Plaat1* deficiency results in differences in heart weights

**Fig. 1:**
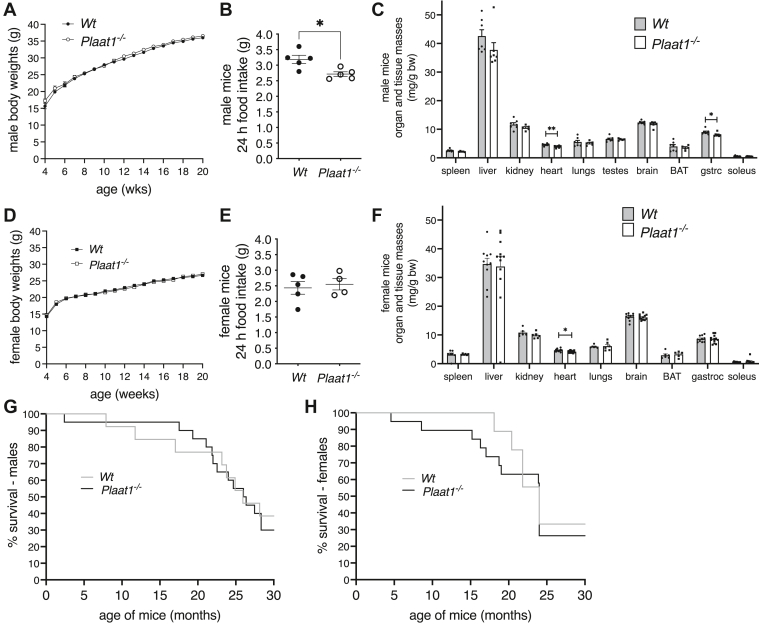
Male and female mouse growth curves, food intakes, tissue weights, and survival. Body weights were monitored from 4 to 20 weeks of age in male (A) and female (D) mice (n = 3–5). Seven-day average food intakes were assessed (n = 4–5) (B, E). Weight-normalized organ and tissue weights of male (n = 5–7) (C) and female (n = 5–12) (F) *Plaat1*^*−/−*^ mice and their control *Wt* littermates. Kaplan–Meier survival curves from weaning until 30 months of age were recorded for male (G) and female (H) mice. Data are means ± SEM. Food intakes, and organ and tissue masses were compared by Student’s *t* test; survival curves were compared using Chi-Square test. ∗*P* < 0.05, ∗∗*P* < 0.01.

Body weights (bw) of *Wt* and *Plaat1*^*−/−*^ mice were essentially indistinguishable when comparisons were made between sex- and age-matched animals (Fig. 1A, D). Interestingly, however, while female mice of both genotypes had similar food intake levels, male *Plaat1*^*−/−*^ mice ate ∼15% less than their *Wt* littermates (Fig. 1B, E). Hearts from male *Plaat1*^*−/−*^ mice were 14.2% smaller than hearts from their *Wt* littermate controls (*Wt*, 4.59 ± 0.14 mg/g bw vs. *Plaat1*^*−/−*^, 3.94 ± 0.14 mg/g bw, *P* = 0.01), while hearts from female *Plaat1*^*−/−*^ mice were 10.6% smaller (*Wt*, 4.68 ± 0.16 mg/g bw vs. *Plaat1*^*−/−*^, 4.19 ± 0.11 mg/g bw, *P* < 0.05) (Fig. 1C, F). In males, gastrocnemius muscles were also 10.2% smaller (*Wt*, 9.01 ± 0.33 mg/g bw vs. *Plaat1*^*−/−*^, 8.10 ± 0.24 mg/g bw, *P* < 0.05) (Fig. 1C). All other organ and tissue weights did not differ statistically in mass between sex-matched *Wt* and *Plaat1*^*−/−*^ mice (Fig. 1C, F).

Longevity was assessed by recording the age of mice at spontaneous death or at ethically mandated euthanasia, from weaning until 30 months of age, and data were recorded in Kaplan–Meier survival curves for males (Fig. 1G) and females (Fig. 1H). Differences between genotypes were analyzed by log-rank test, and no statistically significant alteration in survival was detected in mice deficient in *Plaat1*.

### *Plaat1*^*−/−*^ mice exhibit cardiac cardiolipin deficiency and compositional changes

**Fig. 2:**
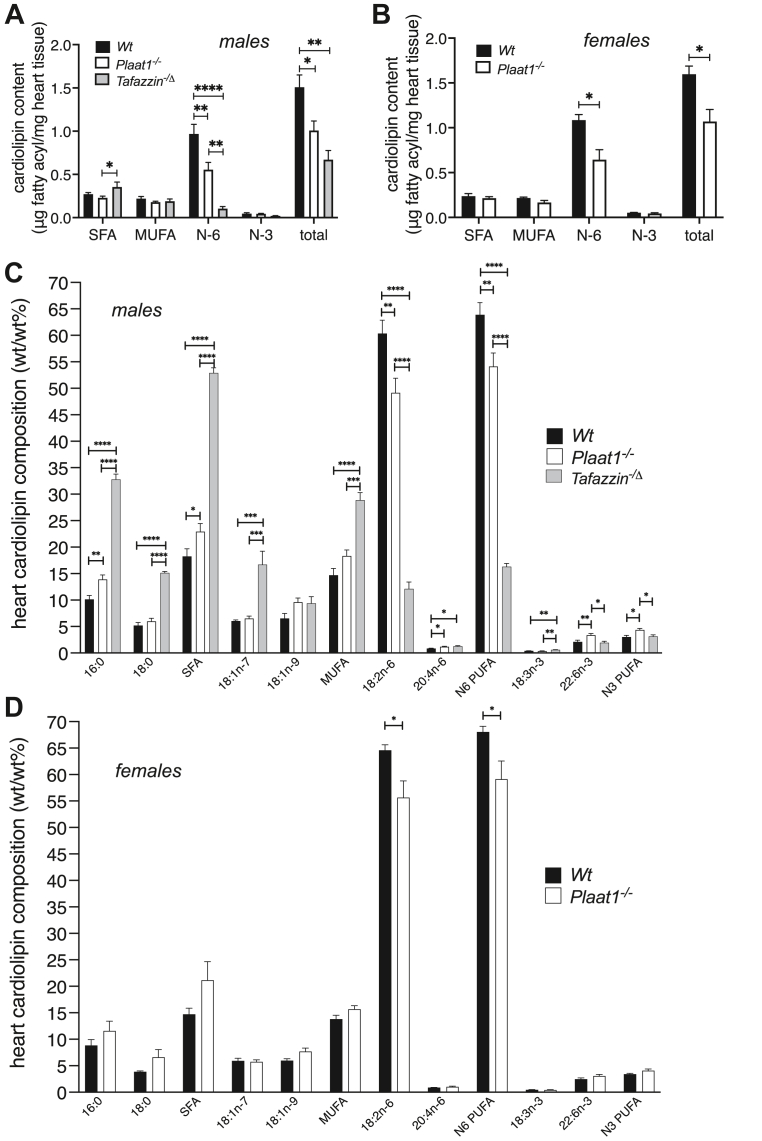
*Plaat1* deficiency alters the concentration and composition of cardiac cardiolipin. Cardiolipin (CL) was isolated from hearts of male and female *Wt* and *Plaat1*^*−/−*^ mice, as well as from male *Tafazzin*^*−/*^^Δ^ mice for comparison, and the fatty acyl content (A, B) was analyzed by gas chromatography. The relative mass percentage (wt/wt%) of major fatty acyl species in cardiac cardiolipin is shown for male (C) and female (D) mice. Data are means ± S.E.M; n = 4–5. ∗*P* < 0.05, ∗∗*P* < 0.01, ∗∗∗*P* < 0.001, ∗∗∗∗*P* < 0.0001.

Remarkable changes were observed in the cardiac cardiolipin content and molecular profile of both female and male *Plaat1*^*−/−*^ mice. In male *Plaat1*^*−/−*^ mice, the total cardiolipin concentration in heart tissue was one-third lower compared to their *Wt* littermates (1.01 ± 0.11 μg cardiolipin fatty acyl/mg tissue vs. 1.51 ± 0.14 μg cardiolipin fatty acyl/mg tissue, *P* < 0.05, respectively) (Fig. 2A, Supplemental Table S1). For comparison, we also analyzed male *Tafazzin*^*−/Δ*^ mice that exhibit a characteristic and profound loss of total cardiolipin in heart tissue due to deficient activity of the Tafazzin cardiolipin remodeling enzyme, which primarily impairs incorporation of linoleoyl (18:2n-6) residues, which are important for the formation of tetralinoleoyl cardiolipin that is enriched in heart tissue (Fig. 2A, Supplemental Table S1) (15, 16, 17). In *Tafazzin*^*−/Δ*^ mice, the cardiac cardiolipin content was 56% lower than in *Wt* mice (0.67 ± 0.11 μg cardiolipin fatty acyl/mg tissue, *P* < 0.01), although this did not differ significantly from the total cardiac cardiolipin content of *Plaat1*^*−/−*^ mice (Fig. 2A). Among the major fatty acid classes analyzed within isolated cardiolipin, only total N-6 polyunsaturated fatty acid (PUFA) concentrations decreased, with male *Plaat1*^*−/−*^ mice exhibiting 43% lower concentrations, and male *Tafazzin*^*−/Δ*^ mice exhibiting 89% lower concentrations than sex-matched *Wt* mice, and 81% lower concentrations than *Plaat1*^*−/−*^ mice, which was largely accounted for by decreases in 18:2n-6 in all cases (Fig. 2A, Supplemental Table S1). Female *Plaat1*^*−/−*^ mice similarly exhibited ∼one-third lower total cardiolipin concentrations in heart tissue compared to their *Wt* littermates (1.07 μg ± 0.13 cardiolipin fatty acyl/mg tissue vs. 1.60 ± 0.09 μg cardiolipin fatty acyl/mg tissue, *P* < 0.05, respectively), and this was also largely due to a 41% lower total N-6 PUFA concentration in the tissue cardiolipin isolates, stemming from reductions in 18:2n-6 that were of a similar magnitude to reductions seen in males (Fig. 2B, Supplemental Table S2).

To compare the relative composition of isolated cardiolipin, fatty acyl masses were expressed as a percentage of the total mass of cardiolipin analyzed within a sample (wt/wt%) (Fig. 2C, D). In males, the relative abundance of total saturated fatty acids (SFA) was significantly higher in *Plaat1*^*−/−*^ mice than their *Wt* littermates, largely as a result of enrichment with palmitate (16:0), while the abundance of total N6 PUFA was significantly lower, largely due to lower levels of 18:2n-6 (Fig. 2C). In this regard, the *Plaat1*^*−/−*^ mouse model was similar to the *Tafazzin*^*−/Δ*^ mouse model, where these changes were also evident, but more pronounced (Fig. 2C). In contrast, male *Plaat1*^*−/−*^ mice had increased docosahexaenoic acid (22:6n-3), which primarily accounted for an elevated abundance of N3 PUFA that was not seen in *Tafazzin* ^*−/*^^Δ^ mice (Fig. 2C). Cardiolipin from male *Tafazzin*^*−/Δ*^ mouse hearts also exhibited a greater abundance of monounsaturated fatty acids (MUFA) that was not evident in *Plaat1*^*−/−*^ mice (Fig. 2C). In female mice, 18:2n-6 and total N6 PUFA were relatively less abundant in *Plaat1*^*−/−*^ mice than *Wt* mice (Fig. 2D), but other major cardiolipin fatty acyl species and classes did not differ significantly between genotypes.

### SDHA is reduced in hearts from *Plaat1*^*−/−*^ mice

Considering the significantly lower content of the mitochondrial signature lipid cardiolipin in both male and female *Plaat1*^*−/−*^ mouse hearts, we performed immunoblotting on lysates from this tissue to quantify relative levels of electron transport chain (ETC) proteins and mitochondrial proteins. These included the ETC Complex I, II, III, and IV proteins NADH:ubiquinone oxidoreductase core subunit S1 (NDUFS1), SDHA, ubiquinol-cytochrome c reductase iron-sulfur subunit (UQCRFS1), and cytochrome c oxidase subunit IV (COX IV), respectively, all of which localize to the inner mitochondrial membrane. It also included heat-shock protein 60 (HSP60), which indirectly supports energy metabolism by acting as a chaperone within the mitochondria where it localizes to the matrix (although it is also found elsewhere in cells) (18), and translocase of the outer mitochondrial membrane 20 (TOM20), which is integral to the outer mitochondrial membrane (19).

**Fig. 3:**
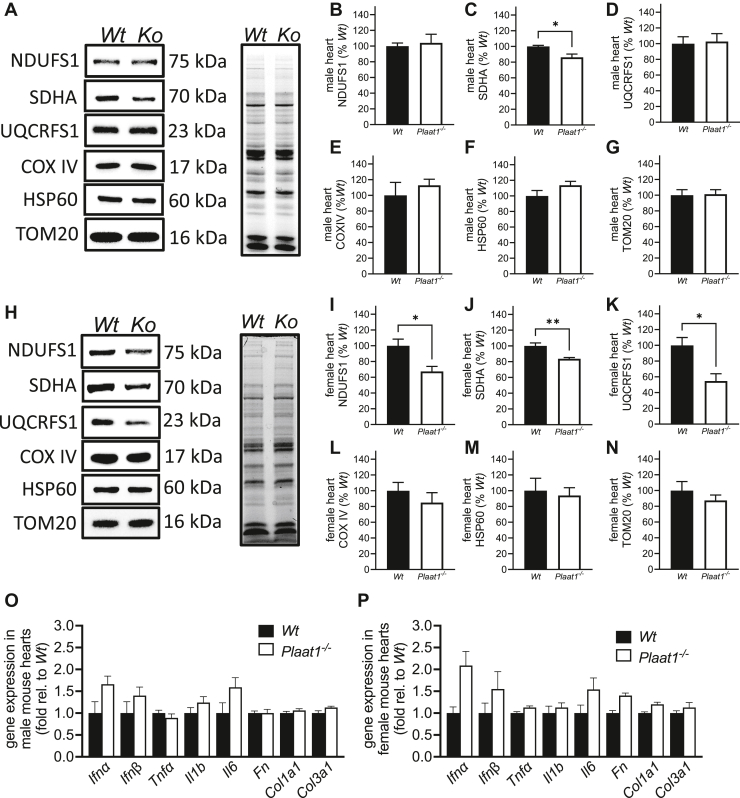
Mitochondrial and electron transport chain protein expression and fibrosis and inflammation gene expression in cardiac tissue. Representative immunoblots from male (A, left hand panel) and female (H, left hand panel) wild-type (*Wt*) and *Plaat1*^*−/−*^ (*Ko*) mouse hearts, along with images of total protein loading (A, H, right hand panels). Quantitation of band densities for NDUFS1 (B, I), SDHA (C, J), UQCRFS1 (D, K), COX IV (E, L), HSP60 (F, M), and TOM20 (G, N) are shown. Results are shown normalized to total protein per lane (A, H right hand panel). Expression of genes involved in inflammation (*Ifn*α*, Ifn*β*, Tnf*α*, Il1b, Il6*) and fibrosis (*Fn, Col1a1, Col3a1*) in male (O) and female (P) hearts. Data are means ± S.E.M (n = 4–5), and comparisons were performed using Student’s *t* test. ∗*P* < 0.05, ∗∗*P* < 0.01.

Surprisingly, in both males and females, COXIV (Fig. 3A, E, H, L), HSP60 (Fig. 3A, F, H, M), and TOM20 (Fig. 3A, G, H, N) were detected at similar levels in *Wt* and *Plaat1*^*−/−*^ mouse hearts. However, SDHA levels were slightly but significantly lower in both male and female *Plaat1*^*−/−*^ hearts by 13.8% and 16.3%, respectively (Fig. 3A, C, H, J). In addition, in female (but not male) heart tissue, NDUFS1 and UQCRFS1 were also significantly lower (Fig. 3A, B, D, H, I, K).

### Inflammation and fibrosis marker gene expression is not significantly elevated in *Plaat1*^*−/−*^mouse hearts

The expression of genes involved in inflammation (*i.e.* interferon alpha (*Ifnα*), interferon beta (*Ifnβ*), tumor necrosis factor alpha (*Tnfα*), interleukin 1b (*Il1b*), and interleukin 6 (*Il6*)), and genes involved in fibrosis (*i.e.* fibronectin (*Fn*), collagen type I alpha1 (*Col1a1*), collagen type III alpha 1 (*Col3a1*)) were analyzed in the hearts of male and female mice by the ΔΔCt method. No significant differences between *Wt* and *Plaat1*^*−/−*^ mice were found (Fig. 3, O, P).

### *Plaat1*^*−/−*^ mice have diminished oxygen consumption, carbon dioxide production, and energy expenditure

**Fig. 4:**
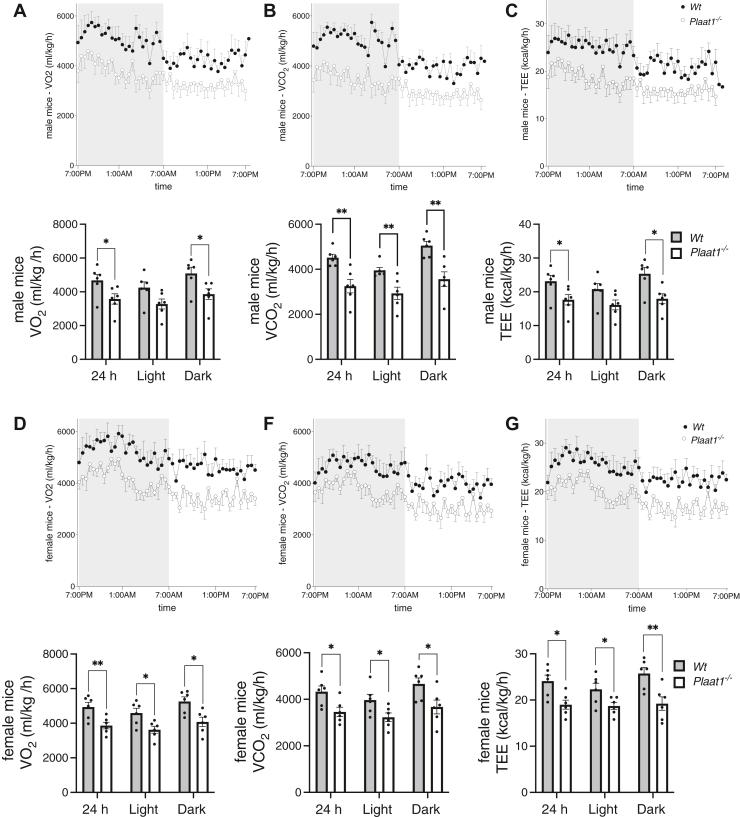
Respiratory gas exchange and energy expenditure. Whole-body VO_2_ (A, D), VCO_2_ (B, E), and TEE (C, F) was determined over a 24-h period in male and female *Plaat1*^*−/−*^ mice and their *Wt* littermates. Line graphs show average measures recorded at 28-min intervals, with the dark period denoted by a shaded background and the light period denoted by an unshaded background. The corresponding bar graphs show calculated averages during the 24 h, light-, and dark-periods. Data are means ± SEM, (n = 6); Statistical comparisons were made by Student’s *t* test. ∗*P* < 0.05, ∗∗*P* < 0.01.

Indirect calorimetry measures were made using the CLAMS system, and metabolic values were analyzed using mean values derived from the 28-min measuring intervals. Male *Plaat1*^*−/−*^ mice displayed lower respiratory gas exchange when compared to *Wt* controls, using 23.6% less oxygen (VO_2_) over the 24-h testing period (*Wt*, 4,680 ± 353 ml/kg/h vs. *Plaat1*^*−/−*^, 3,576 ± 305 ml/kg/h, *P* < 0.05) (Fig. 4A). Mean differences between groups in the light phase did not reach statistical significance (*P* = 0.0599), but in the dark phase, PLAAT1-deficient mice consumed an average of 24.1% less oxygen (*Wt*, 5,092 ± 363 ml/kg/h vs. *Plaat1*^*−/−*^, 3,863 ± 316 ml/kg/h, *P* < 0.05) (Fig. 4A). Similarly, 28% lower VCO_2_ was also noted in the male *Plaat1*^*−/−*^ mice (Fig. 4B) over the 24-h overall reading (*Wt*, 4,518 ± 152 ml/kg/h versus *Plaat1*^*−/−*^, 3,254 ± 300 ml/kg/h, *P* < 0.01). However, statistically significant reductions of 25.8% and 29.5% in CO_2_ production, respectively, were evident in both the 12-h light (*Wt*, 3,952 ± 129 ml/kg/h versus *Plaat1*^*−/−*^, 2,934 ± 276 ml/kg/h, *P* < 0.01), and dark phases (*Wt*, 5,055 ± 190 ml/kg/h versus *Plaat1*^*−/−*^, 3,562 ± 325 ml/kg/h, *P* < 0.01), in *Plaat1*^*−/−*^ male mice (Fig. 4B). TEE was 23.7% lower in male *Plaat1*^*−/−*^ over the 24-h period (*Wt*, 23.13 ± 1.76 kcal/kg/h versus *Plaat1*^*−/−*^, 17.65 ± 1.52 kcal/kg/h, *P* < 0.05) (Fig. 4C). Although differences in the light phase did not reach statistical significance (*P* = 0.06), mean TEE was 29.1% lower for *Plaat1*^*−/−*^ mice in the dark phase (*Wt*, 25.34 ± 1.86 kcal/kg/h versus *Plaat1*^*−/−*^, 17.96 ± 1.47 kcal/kg/h, *P* < 0.05) (Fig. 4C).

Overall, female mice displayed similar patterns of differences between genotypes in these metabolic parameters. Average oxygen consumption over the 24-h testing period was 21.8% lower in female *Plaat1*^*−/−*^ mice compared to their *Wt* littermates (*Wt*, 4,937 ± 267 ml/kg/h versus *Plaat1*^*−/−*^, 3,862 ± 195 ml/kg/h, *P* < 0.01) (Fig. 4D). In the light period, PLAAT1-deficient female mice consumed 21% less oxygen during the light phase (*Wt*, 4,589 ± 273 ml/kg/h versus *Plaat1*^*−/−*^, 3,624 ± 201 ml/kg/h, *P* < 0.05), and 22.4% less oxygen during the dark phase (*Wt*, 5,258 ± 263 ml/kg/h versus *Plaat1*^*−/−*^, 4,080 ± 268 ml/kg/h, *P* < 0.01) (Fig. 4D). VCO_2_ was also lower in the female *Plaat1*^*−/−*^ mice, and this was noted over the combined 24-h period (*Wt*, 4,328 ± 244 ml/kg/h versus *Plaat1*^*−/−*^, 3,459 ± 201 ml/kg/h, *P* < 0.05), and in the light (*Wt*, 4,003 ± 246 ml/kg/h versus *Plaat1*^*−/−*^, 3,083 ± 194 ml/kg/h, *P* < 0.05, and dark phases (*Wt*, 4,663 ± 254 ml/kg/h versus *Plaat1*^*−/−*^, 3,676 ± 296 ml/kg/h, *P* < 0.05), where decreases of 20.1%, 23%, and 21.2% were observed, respectively, (Fig. 4E). Female *Plaat1*^*−/−*^ mice had lower TEE compared to their *Wt* littermates, which was 21.2% lower over the 24-h period (*Wt*, 24.10 ± 1.29 kcal/kg/h versus *Plaat1*^*−/−*^, 18.99 ± 0.98 kcal/kg/h, *P* < 0.05), 16.2% lower during the light phase (*Wt*, 22.32 ± 1.29 kcal/kg/h versus *Plaat1*^*−/−*^, 18.70 ± 0.77 kcal/kg/h, *P* < 0.05), and 25.3% lower over the dark phase (*Wt*, 25.74 ± 1.30 kcal/kg/h versus *Plaat1*^*−/−*^, 19.22 ± 1.47 kcal/kg/h, *P* < 0.01) (Fig. 4F).

**Fig. 5:**
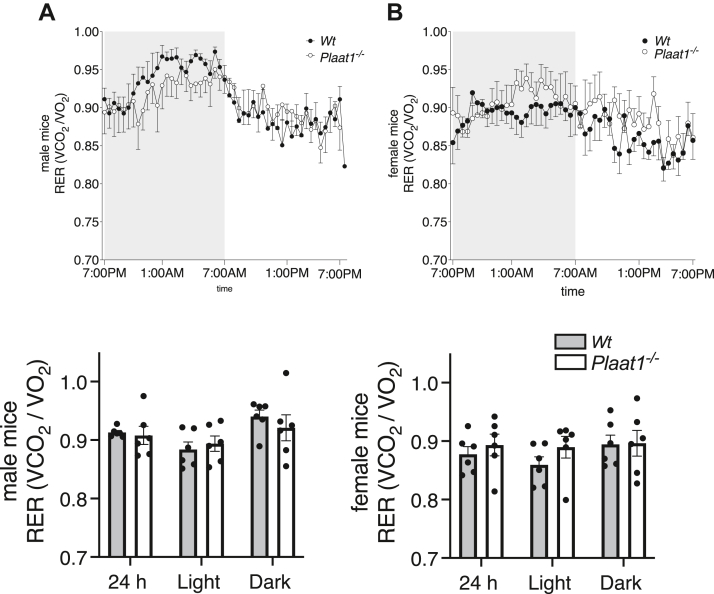
RER measures in male and female mice. RERs were calculated for male (A) and female (B) mice and are shown in upper line graphs as average measures assessed at 28-min intervals over 24 h, with the dark photoperiod denoted by shaded background, and the light photoperiod denoted by the unshaded background. Bar graphs depicting 24 h, dark-period, and light-period averages are also shown. Data are means ± SEM (n = 6).

RER were calculated for male (Fig. 5A) and female (Fig. 5B) mice but did not differ significantly between genotypes over the course of the 24-h period analyzed, or during either the light- or dark-phases.

### Male *Plaat1*^*−/−*^ mice have lower rearing activity

**Fig. 6:**
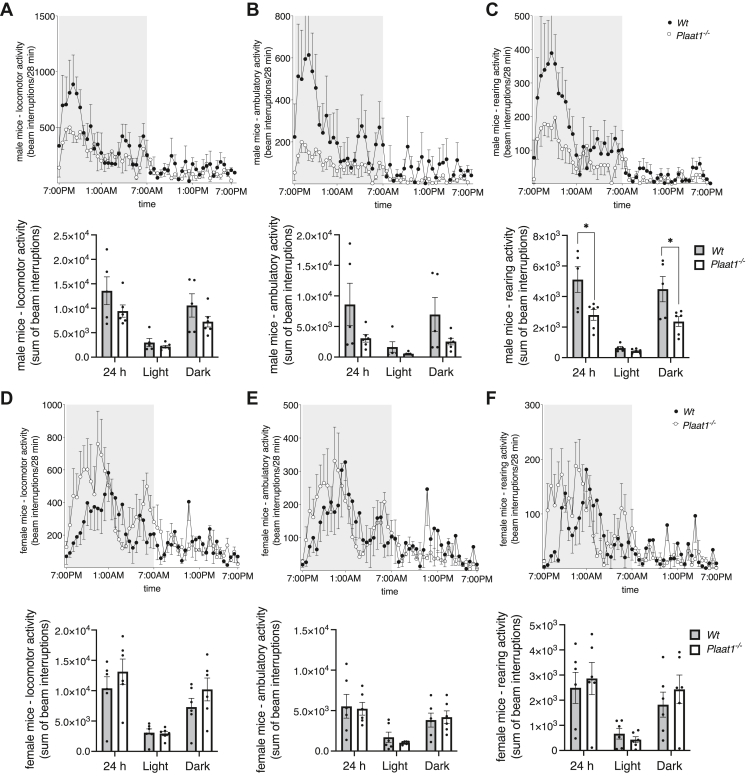
Voluntary activity. Sums of infrared (IR) beam interruptions were recorded at 28 min intervals over a 24 h period for male and female *Plaat1*^*−/−*^ and littermate *Wt* mice resulting from locomotion (A, D), ambulation (B, E), and rearing (C, F). Movement in the dark period is denoted by shaded background, while movement in the light period is denoted by an unshaded background. The corresponding bar graphs show total sums of IR beam interruptions during the 24 h period, 12 h dark period, and 12 h light period. Data are means ± SEM (n = 5–6). ∗*P* < 0.05.

The CLAMS apparatus measures various forms of activity such as infrared (IR) beam interruptions, including ambulation movements, such as when a mouse traverses a chamber, total locomotion movements, such as grooming and feeding in addition to ambulation, and rearing movements. Locomotion and ambulation did not differ significantly between either male or female *Plaat1*^*−/−*^ mice and their sex-matched *Wt* littermates over the 24-h period analyzed, or during either the 12-h light or dark phases when analyzed in aggregate (Fig. 6A, B, D, E). Total rearing activity by male *Plaat1*^*−/−*^ mice was 45.3% lower over a 24-h period (*Wt*, 5,111 ± 839 versus *Plaat1*^*−/−*^, 2,793 ± 352 total IR beam interruptions, *P* < 0.05), and was 47.3% lower during the dark phase (*Wt*, 4,483 ± 826 versus *Plaat1*^*−/−*^, 2,364 ± 321 IR beam interruptions, *P* < 0.05) (Fig. 6C), although measures did not differ significantly in the light phase. Female mice did not display statistically significant differences between genotypes in rearing activity during the 24 h, light- or dark-periods (Fig. 6F).

### *Plaat1*^*−/−*^ mice have lower exercise endurance

**Fig. 7:**
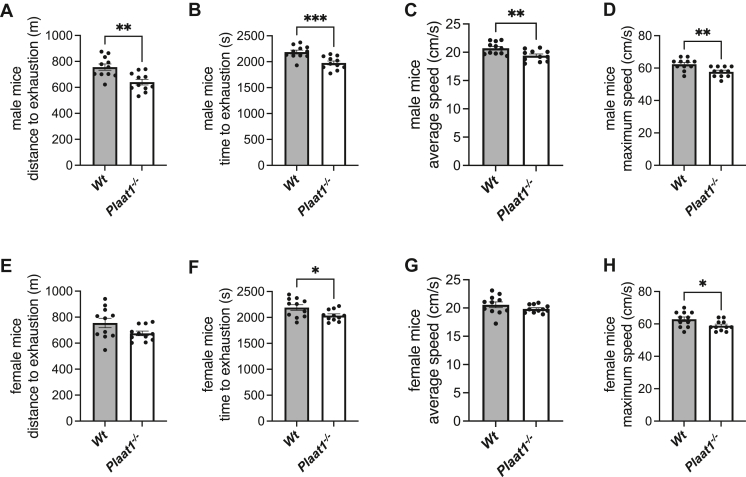
Exercise capacity. Using a treadmill test, the distance that male and female mice were able to run until exhaustion (A, E), the time at exhaustion (B, F), the average speed over the test period (C, G), and the maximum speed reached at exhaustion (D, H) were recorded. Data are means ± SEM (n = 11). ∗*P* < 0.05, ∗∗*P* < 0.01, ∗∗∗*P* < 0.001.

Maximum exercise capacity was assessed using a motorized treadmill performance test. Male *Plaat1*^*−/−*^ mice exhibited significantly lower exercise capacity based on all measures recorded. Male *Plaat1*^*−/−*^ mice ran a 15.3% shorter distance before exhausting than their sex-matched *Wt* littermates (640.4 ± 21.4 m versus 756.5 ± 24.2 m, respectively, *P* < 0.01) (Fig. 7A). This was reflected also in the earlier time to exhaustion for *Plaat1*^*−/−*^ mice, that were able to run an average of only 1,976 ± 37.2 s, compared to male *Wt* mice that ran 2,186 ± 37.4 s, *P* < 0.001 (Fig. 7B). Male *Plaat1*^*−/−*^ mice had a 6.3% lower average speed over the course of the run (Fig. 7C) and 7.4% lower average maximal speed achieved by the end of the run (Fig. 7D), compared to their *Wt* littermates.

Female mice also exhibited deficiencies in exercise endurance. While the distance to exhaustion did not differ significantly between female *Wt* and *Plaat1*^*−/−*^ mice (*P* = 0.06) (Fig. 7E), female *Plaat1*^*−/−*^ mice did exhaust 7.0% faster than their *Wt* littermates (2,037 ± 32.3 s versus 2,191 ± 54.4 s, respectively, *P* < 0.05) (Fig. 7F). Average speeds achieved over the course of the test were not significantly different between female mice of different genotypes (Fig. 7G). However, the maximal speed that could be obtained at the end of the trial was 6.4% lower in female *Plaat1*^*−/−*^ mice that achieved only 58.9 ± 1.0 cm/s, compared to female *Wt* mice that achieved 62.9 ± 1.4 cm/s (*P* < 0.05) (Fig. 7H).

## Discussion

To better understand the physiological role of *Plaat1*, we generated mice deficient in this enzyme, and studied their gross morphology, various metabolic and activity-related characteristics, lifespan, and exercise tolerance. Based on our recent findings of a function for PLAAT1 in the remodeling of cardiolipin in vitro (2), and prior reports of enrichment of this enzyme in heart tissue (2), we also focused our study on the content and composition of cardiac cardiolipin in these mice. Interestingly, although the phenotype of our *Plaat1*^*−/−*^ mice initially appeared grossly unremarkable, careful evaluation revealed interesting and significant molecular and physiological differences.

Our study demonstrates that *Plaat1* is a critical regulator of cardiac cardiolipin content. This tissue exhibited striking differences between *Wt* and *Plaat1*^*−/−*^ mice, with total cardiolipin concentrations measuring approximately a third lower in both male and female knockout mice. Further assessment indicated that this difference was largely due to a decline in the relative and total contents of n-6 PUFA, with both male and female mice exhibiting significantly lower levels of 18:2n-6. While not as drastic as the reduction in total cardiolipin 18:2n-6 content observed in male *Tafazzin*^*−/Δ*^ mice, this decline was still quantitatively significant. Also, similar to *Tafazzin*^*−/Δ*^ mice, male *Plaat1*^*−/−*^ mice had higher relative levels of palmitate, which could indicate compensation from increased de novo synthesis of cardiolipin, although additional studies will be required to better understand the molecular and cellular basis for these changes.

In this regard, PLAAT1 is known to catalyze multiple reactions, and therefore the contributions of this enzyme to cardiolipin content and composition in vivo are likely to be multi-faceted and may involve direct and/or indirect effects. Indeed, comparison of data from our prior work on the PC:MLCL transacylase activity of PLAAT1 in vitro (2) with data from the current study suggests that alterations in knockout mouse cardiac cardiolipin may more likely result from a loss of the indirect activity (or activities) of PLAAT1 that *influence* cardiolipin levels, than the direct action of this enzyme on cardiolipin remodeling, per se. For example, our prior analyses of the action of PLAAT1 in vitro and in cells suggests a preference of this enzyme for saturated and monounsaturated fatty acyl chains, yet loss of *Plaat1* in the current study was not associated with lower SFA or MUFA enrichment (2). Instead, there was a loss of linoleate. Since tetra-linoleoyl cardiolipin is highly enriched in cardiac tissue, our findings likely reflect the lower total levels of cardiolipin in knockout mouse hearts (20, 21), rather than specific impairments in remodeling, per se. Regardless, the current work does not directly explain the mechanism underlying the drastic decline in cardiac cardiolipin observed with PLAAT1 loss, and given the likely complexity, considerable additional study is expected to be required to elucidate this. Thus, although our previous and current findings together could suggest a direct role for PLAAT1 in cardiac cardiolipin remodeling, it is important that our results are also considered in the context of additional known enzymatic functions of the PLAAT1 enzyme.

Prior research has shown that PLAAT1 displays PLA1/2 activity (3). This can alter the liberation of bioactive fatty acids, affecting downstream pathways and processes, sometimes far removed from the initial effect. Indeed, impaired adipocyte triacylglycerol storage in *Plaat3* knockout mice is caused by reduced arachidonic acid generation from membrane phospholipids following loss of this PLA1/2, which in turn lowers levels of derivative prostaglandins, to then remove the inhibitory “braking” signal of these compounds on lipolysis, leading to excessive fat breakdown (22).

In addition, PLAAT1 also has N-transacylase activity, and therefore can increase the vivo synthesis of NAPE (N-acylphosphatidylethanolamine) when over-expressed in cells (3, 23). NAPE are the immediate precursors for the generation of N-acylethanolamide (NAE) signaling molecules that can elicit a host of effects in various biological systems (24), and the rise in PLAAT1-mediated NAPE that occurs in cells overexpressing this enzyme is, indeed, associated with an increase in the cellular content of NAE (25). NAE are agonists of several receptor proteins that can affect mitochondria (26).

Of relevance to the current work, the NAE oleoylethanolamide (OEA) is a high-affinity ligand for peroxisome proliferator-activated receptor alpha (PPARα), which has been shown to signal in phospholipid synthesis in murine tissues (27). PPARα activation can induce the expression of lysophosphatidic acid acyltransferase (LPAAT) enzymes that catalyze the synthesis of PA in the Kennedy Pathway from which all complex lipids, including nascent cardiolipin, are produced (28). PPARα activation has also been shown to stimulate de novo cardiolipin biosynthesis via an increase in phosphatidylglycerol phosphate (PGP) synthase activity (29), catalyzing the conversing of cytidine diphosphate (CDP)- diacylglycerol to PGP, which comprises the penultimate step for the synthesis of nascent cardiolipin (30). Whether changes in these enzyme systems, or in bioactive NAE concentrations or transcription factor activation occur in the hearts of *Plaat1*^*−/−*^ mice were not studied in the current work. Significant additional study will be required to elucidate the relative extent to which direct effects of PLAAT1 on cardiolipin synthesis and remodeling, as well as indirect effects of this enzyme on lipid signaling pathways, among others, contribute to the cardiolipin changes seen in *Plaat1*-deficient cardiac tissue.

A lower content of cardiac cardiolipin could indicate that the concentration of this lipid in mitochondria is reduced, or that there has been a decline in the relative abundance of mitochondria. In other models of cardiolipin deficiency, such as the *Tafazzin*^*−/Δ*^ model, a compensatory upregulation of mitochondria, with a relative deficiency in cardiolipin per mitochondrion, has been reported (20). Immunoblotting for the mitochondrial matrix protein HSP60, the outer mitochondrial membrane protein TOM20, and the ETC Complex IV protein COX IV indicated no significant differences in heart with *Plaat1* loss in male or female mice. However, small but significant reductions in cardiac SDHA levels were detected. As an integral component of the inner mitochondrial membrane, cardiolipin is known to engage with electron transport chain (ETC) complexes, maintaining their structural integrity and optimal enzymatic function (31, 32, 33, 34). Distinct binding locations for cardiolipin within complexes I, III, and IV have been identified (32). Although a binding site for cardiolipin to complex II, which contains SDHA, has not been reported (32), it has been shown that cardiolipin is essential for ensuring the optimal stability, assembly, and enzymatic function of this complex (35). Interestingly, lower levels of the ETC Complex I protein NDUFS1 and Complex III protein UQCRFS1 were also detected in hearts of *Plaat1* deficient female mice, but this difference was not seen in male mice, indicating a sexual dimorphism in the phenotype of the mice. The reason for this difference was not apparent from the current data but notably cannot be explained by differences in total cardiac cardiolipin, since levels were similarly reduced in male and female mice.

The significance of these findings with regard to the direct function of cardiac mitochondrial complexes I, II and III in *Plaat1*^*−/−*^ mice remains to be determined. Notably, though, the content of several ETC proteins, including UQCRFS1, SDHA, and two additional NDUF subunit proteins are reportedly also lower in cardiolipin-deficient *Tafazzin* knockdown mouse models (36, 37). SDHA, which was affected in both male and female hearts, is of interest in that it is a structural subunit of complex II, and is functional in both the TCA cycle and ETC, participating in both processes at the interface between them (38, 39). In the TCA cycle, SDHA catalyzes the oxidation of succinate to fumarate, and this reaction is coupled with the reduction of flavin adenine dinucleotide (FAD) to FADH_2_ (38, 39, 40). The electrons from FADH_2_ generated by SDHA are then transferred to the electron transport chain at complex II (38, 39, 40). Thus, it is plausible that diminished cardiolipin levels in *Plaat1*^*−/−*^ mouse hearts could have contributed to the reduced cardiac SDHA content due to diminished stability, while the lack of change in HSP60 and COXIV could suggest that the overall mitochondrial content is not significantly altered. Future studies to examine mitochondrial ultrastructure using electron microscopy are planned to better understand the nature of changes arising in cardiac tissue. Regardless, impaired bioenergetic function is a likely consequence, and may be more pronounced in females than in males.

To gain additional insight into the physiological role of PLAAT1, measures of body and organ masses, food intakes, respiration, activity, and exercise capacity were carried out. Many of these measures integrate inputs from multiple body units, including the brain and skeletal muscle, in addition to the cardiovascular system, all of which express *Plaat1* and are predicted to be affected in complex and interacting ways by loss of this enzyme. Thus, significant additional work will be needed to isolate tissue- and organ-specific effects of this enzyme. However, our work uncovered several interesting effects associated with the whole-body loss of *Plaat1*.

All life processes, including the growth and maturation of tissues, require energy. It was therefore surprising that body weights were remarkably similar overall between *Wt* and *Plaat1*^*−/−*^ mice when comparisons were made within sex-matched littermates, although heart weights were lower in both male and female *Plaat1*^*−/−*^ mice, and gastrocnemius muscle depots were also smaller in male mice deficient in this enzyme. Rahman *et al.* similarly reported that skeletal muscle weights were lower in male *Plaat1*^*−/−*^ mice fed a standard diet, and they also observed lower liver weights when mice were challenged with a high-fat diet, although they did not assess heart masses (41). Our findings indicate that deficiency of *Plaat1* does not significantly alter lifespan in either sex. It does, however, significantly alter respiration.

Female and male *Plaat1*^*−/−*^ mice had lower oxygen utilization and VCO_2_, accompanied by diminished energy expenditure, compared to their *Wt* littermate controls. Male *Plaat1*^*−/−*^ mice appeared to compensate for this reduced energetic activity by eating less, and exhibiting lower levels of rearing activity, which likely contributed to their ability to maintain body weights that closely approximated those of their *Wt* littermates despite lower whole-body respiratory substrate use. Male *Tafazzin*^*−/Δ*^ mice similarly show some evidence of reduced rearing activity but in contrast have higher weight-normalized food intakes and respiratory exchange rates, indicating reduced metabolic efficiency (6).

Female *Plaat1*^*−/−*^ mice differed from males in that they had similar food intakes and voluntary activity levels to those of their sex-matched *Wt* littermates, despite lower use of respiratory substrates. While this should create a bioenergetic imbalance, weight gain was not observed. As higher rates of activity, which could offset a fuel surplus, were also not evident, it is unclear how overall energy balance resulting in body weight maintenance was achieved by female *Plaat1*^*−/−*^ mice at levels similar to their *Wt* controls. Future studies to evaluate effects of *Plaat1* deficiency on the absorptive capacity of these animals may provide mechanistic insight. Notably, respiratory exchange ratios were similar between *Wt* and *Plaat1*^*−/−*^ mice, indicating that the predominant types of fuels used were similar in composition.

Given the significant difference in cardiac cardiolipin contents and respiratory measures, we evaluated the effect of *Plaat1* deficiency on maximal exercise capacity in male and female mice. Significant deficits were evident in both sexes. Male *Plaat1*^*−/−*^ mice exhibited reduced performance relative to *Wt* littermates on all metrics evaluated, exhausting after a shorter distance and time, and achieving lower average and maximal speeds. Female *Plaat1*^*−/−*^ mice exhausted earlier than their *Wt* littermates and achieved a lower final maximal speed, which reflects the earlier termination of this progressive test. These results are similar to those reported in male *Tafazzin*^*−/Δ*^ mice, where significant deficits in all aspects of exercise performance were reported, as well as female mice heterozygous for loss of *Tafazzin*, that also had lower maximal speed attainment (6, 10).

Overall, this study demonstrates an important role for PLAAT1 in heart weight and cardiac cardiolipin content, with implications for regulating energy metabolism and exercise capacity. To the best of our knowledge, this is the first study to examine the effects of *Plaat1* deficiency on tissue cardiolipin levels in vivo, or on physiological measures in female animals. A role for PLAAT1 in the regulated degradation of mitochondria and other organelles in the crystalline lens of the eye has also recently been indicated, although a role for this enzyme in cardiolipin metabolism was not examined (42). Future work will explore the molecular basis for alterations in cardiac cardiolipin content and composition as well as effects on mitochondrial structure and function in this organ.

## Data availability

Data are available upon reasonable request to the authors.

## Supplemental data

This article contains supplemental data.

## Conflict of interest

The authors declare the following financial interests/personal relationships, which may be considered as potential competing interests: K.D.S. is currently on the Board of Directors of the International Society for the Study of Fatty Acids and Lipids. None of the other authors have any conflicts of interest to disclose. The funders had no role in the design of the study; in the collection, analyses, or interpretation of data, or in the writing of the manuscript or decision to publish the results
