# Supplementary material for: Plaat1 deficiency reduces cardiac cardiolipin content and impairs exercise tolerance
Source: J Lipid Res. 2025 May 8;66(6):100822. doi: 10.1016/j.jlr.2025.100822 (PMC12169743; doi:10.1016/j.jlr.2025.100822)
Supplement: Appendix 1 [file mmc1.pdf]

Appendix 1

A)

Exon 2: MAVNDCFSLTYPHNPHPGDLIEVFRPCYQHWALYLGDGYVINIAPI

Exon 3: DGIRSSFTSAKSVFSTKALVKMQLLKDVVGNDTYRINNKYDTTYP

PLPVEEVIQRSEFAIGQEVAYDLLVNNCEHFVTLLRYGEGVSEQ

Exon 4: ANRAIGTIGLVAAGIDIFTFLGLFPKRQRTKY

B)

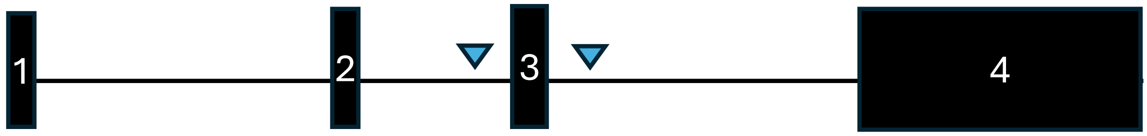

sgRNA target sites used to generate *knockout* of exon 3

NM_013751.7
NP_038779.2
NC_000082.7 Chromosome 16 Reference GRCm39 C57Bl/6J

C)

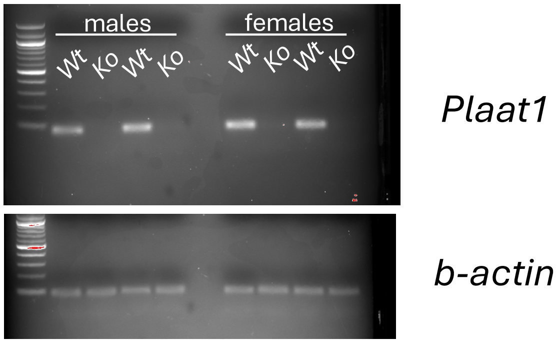

Supplementary Figure 1: The sequence of the PLAAT1 protein, organized into the three exonic coding regions, is shown in (A). Exon 3 (in bold) contains a catalytically critical cysteine residue (amino acids in the catalytic triad are highlighted in blue), and this cysteine occurs in a region (underlined) that is highly conserved within the PLAAT1 family and across species. The predicted C-terminal transmembrane domain is highlighted in yellow.

To generate *Plaat1*$^{-/-}$ mice, exon 3 of the *Plaat1* gene on Chromosome 16 was deleted in C57Bl/6J zygotes using CRISPR by technicians at The Centre for Phenogenomics (TCP), Toronto, ON, Canada, producing *Plaat1*$^{+/-}$ (heterozygous) mice that were bred to generate mice homozygous for the recombined allele, along with their *wildtype* littermates. Loss of exon 3 was confirmed by direct sequencing which identified recombination between segments in intron 2 and 3, denoted by blue arrows where small guide RNA (sgRNA) directed strand cuts as illustrated in (B).

PCR amplification of cDNA generated from hearts using primers targeting a 78 bp region within exon 3 (forward 5’-ggg cag gaa gta gcc tat gac-3’; reverse 5’-cac tcc ttc tcc ata gcg ca) generates amplicons in wildtype (*Wt*) mice are absent in *Plaat1*$^{-/-}$ (*Ko*) mice, although both groups generated a band using primers for beta-actin (forward 5’-cag cca ctg tcg agt-3’; reverse 5’-tcc atg gcg aac tgg t-3’).

Appendix 1

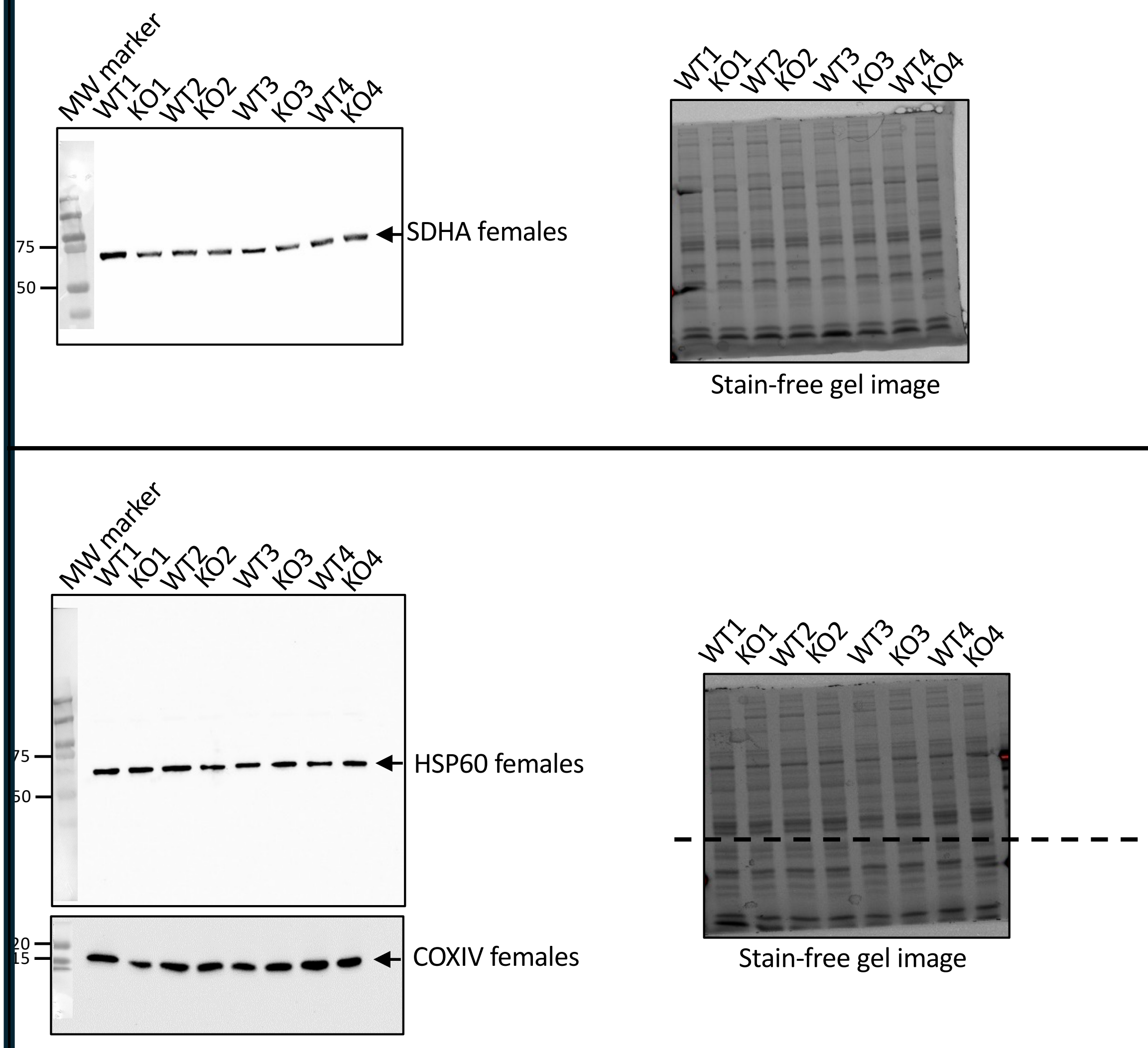

Supplementary Figure 2: Uncropped and unadjusted immunoblots of SDHA, HSP60 and COXIV protein in cardiac tissue from female *Wt* (WT) and *Plaat1* knockout mice (KO) (n=4). The corresponding stain-free™ gel imaged under ultraviolet transillumination demonstrating total protein loading in each lane is shown in the image to the right of each immunoblot. Molecular weight markers were imaged under visual light, and are shown superimposed on the blot in the first lane. Dashed lines show where the blot was cut prior to probing.

Appendix 1

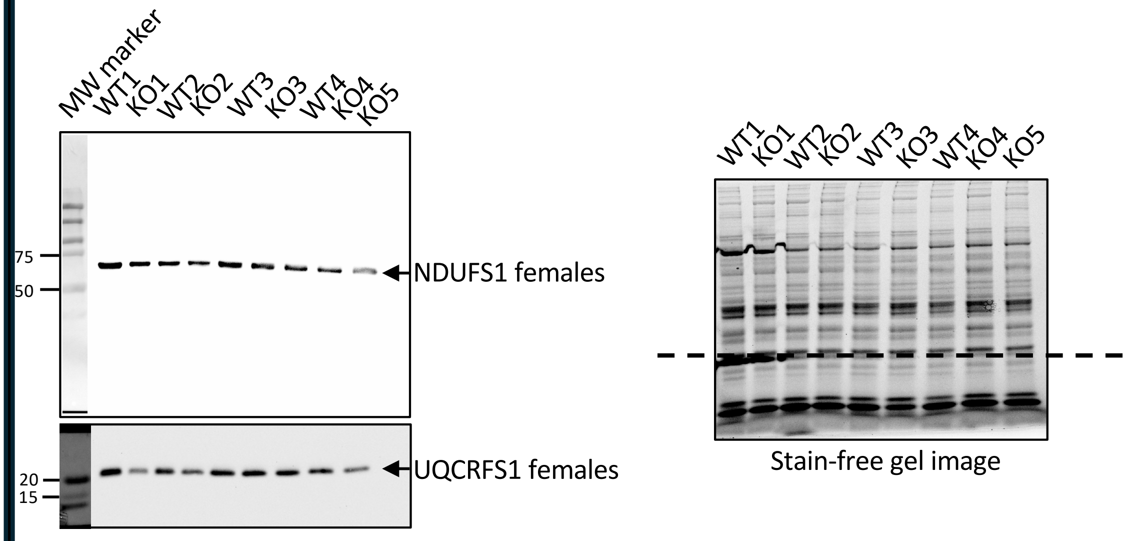

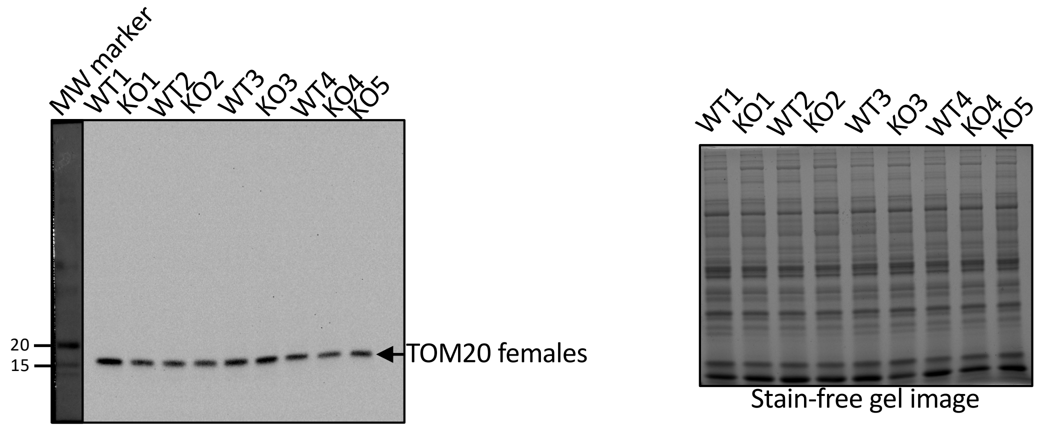

Supplementary Figure 3: Uncropped and unadjusted immunoblots of NDUFS1, UQCRFS1 and TOM20 protein in cardiac tissue from female *Wt* (WT) and *Plaat1* knockout mice (KO) (n=4). The corresponding stain-free™ gel imaged under ultraviolet transillumination demonstrating total protein loading in each lane is shown in the image to the right of each immunoblot. Molecular weight markers were imaged under visual light, and are shown superimposed on the blot in the first lane. Dashed lines show where the blot was cut prior to probing.

Appendix 1

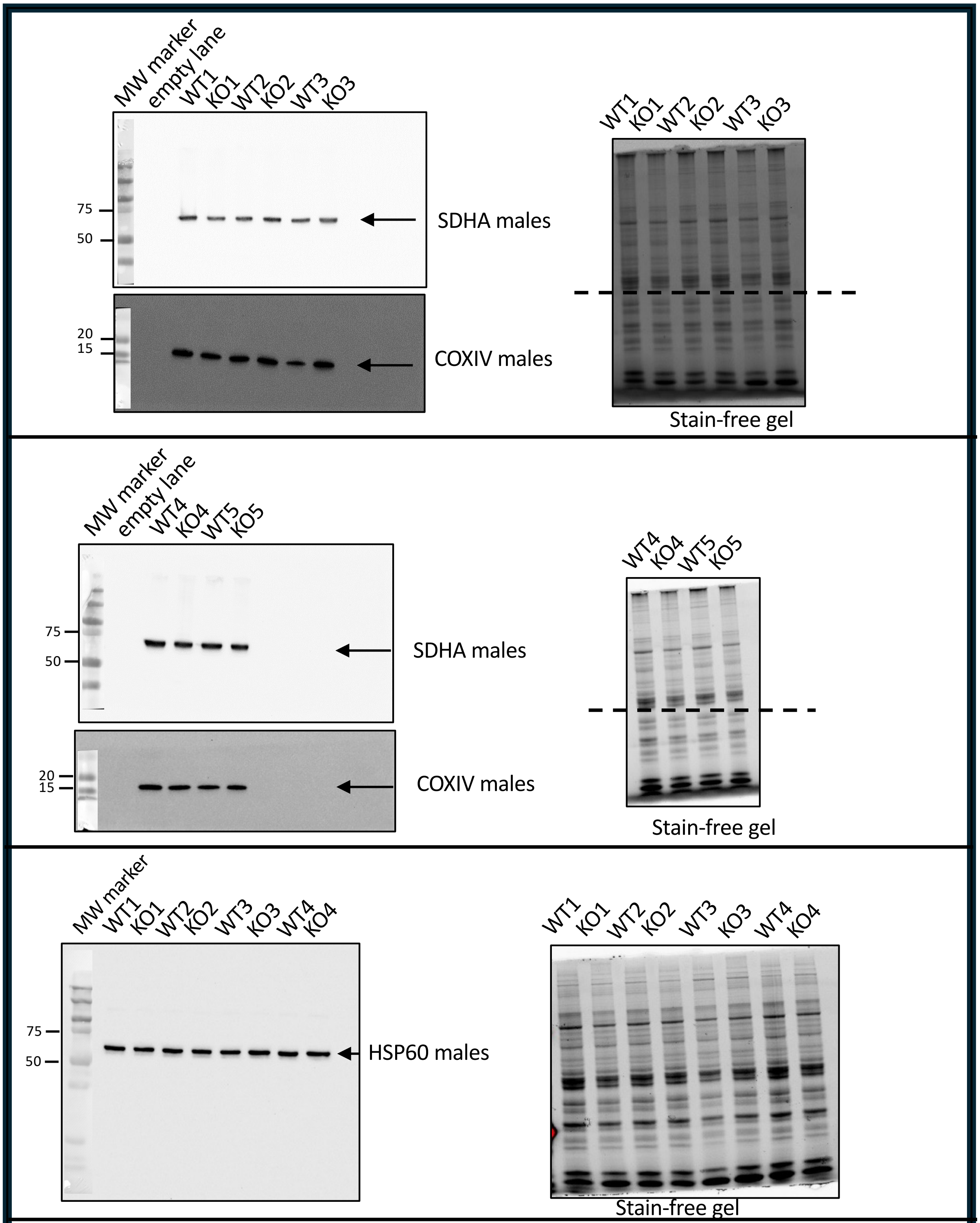

Supplementary Figure 4: Uncropped and unadjusted immunoblots of SDHA, HSP60 and COXIV protein in cardiac tissue from male *Wt* (WT) and *Plaat1* knockout mice (KO) (n=4-5). The corresponding stain-free™ gel imaged under ultraviolet transillumination demonstrating total protein loading in each lane is shown in the image to the right of each immunoblot. Molecular weight markers were imaged under visual light, and are shown superimposed on the blot in the first lane. Dashed lines show where the blot was cut prior to probing.

Appendix 1

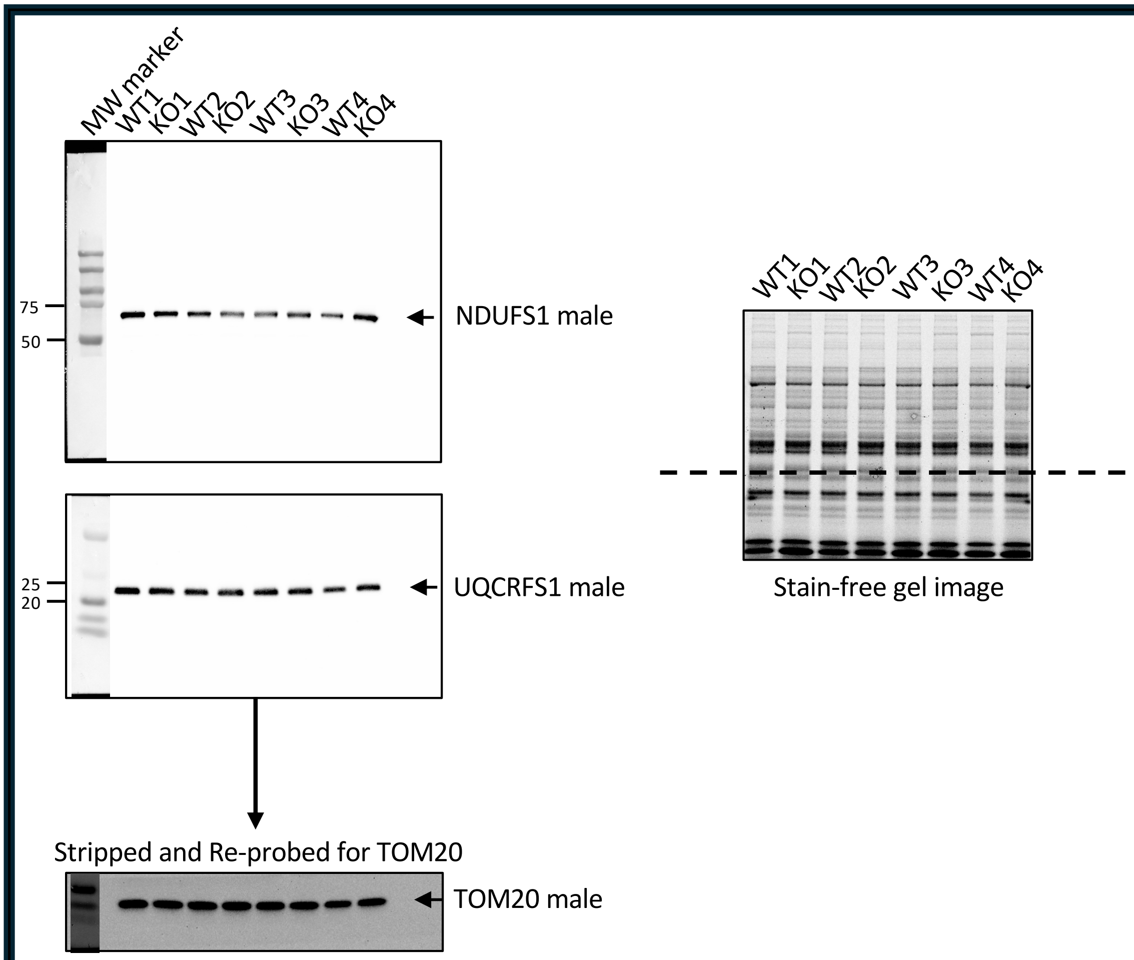

Supplementary Figure 5: Uncropped and unadjusted immunoblots of NDUFS1, UQCRFS1 and TOM20 protein in cardiac tissue from female *Wt* (WT) and *Plaat1* knockout mice (KO) (n=4). The corresponding stain-free™ gel imaged under ultraviolet transillumination demonstrating total protein loading in each lane is shown in the image to the right of each immunoblot. Molecular weight markers were imaged under visual light and are shown superimposed on the blot in the first lane. Dashed lines show where the blot was cut prior to probing.

Supplementary Table 1. Concentrations of cardiolipin fatty acyl species in heart tissue from male *Wt*, *Plaat1*$^{-/-}$, and *Tafazzin*$^{-/\Delta}$ mice.

cardiolipin fatty acyl species	*Wt* (µg/mg of tissue)	*Plaat1*$^{-/-}$ (µg/mg of tissue)	*Tafazzin*$^{-/\Delta}$ (µg/mg of tissue)	*Wt vs Plaat1*$^{-/-}$	*Wt vs Tafazzin*$^{-/\Delta}$	*Plaat1*$^{-/-}$ *vs Tafazzin*$^{-/\Delta}$
10:0	0.0002 ± 0.0000	0.0003 ± 0.0001	0.0003 ± 0.0001			
12:0	0.0041 ± 0.0001	0.0029 ± 0.0006	0.0017 ± 0.0005			
14:0	0.0087 ± 0.0005	0.0074 ± 0.0007	0.0080 ± 0.0017			
15:0	0.0160 ± 0.0027	0.0072 ± 0.0008	0.0067 ± 0.0012	**	*	
16:0	0.1521 ± 0.0144	0.1373 ± 0.0181	0.2206 ± 0.0385			
17:0	0.0068 ± 0.0004	0.0063 ± 0.0008	0.0070 ± 0.0009			
18:0	0.0755 ± 0.0051	0.0590 ± 0.0061	0.1014 ± 0.0157			*
20:0	0.0027 ± 0.0003	0.0021 ± 0.0005	0.0028 ± 0.0004			
22:0	0.0016 ± 0.0005	0.0025 ± 0.0006	0.0038 ± 0.0013			
23:0	0.0008 ± 0.0002	0.0008 ± 0.0003	0.0010 ± 0.0002			
24:0	0.0017 ± 0.0009	0.0010 ± 0.0003	0.0005 ± 0.0002			
Total SFA	0.2720 ± 0.0188	0.2290 ± 0.0200	0.3550 ± 0.0560			*
12:1	0.0006 ± 0.0002	0.0008 ± 0.0002	0.0005 ± 0.0002			
14:1	0.0009 ± 0.0002	0.0009 ± 0.0002	0.0007 ± 0.0001			
16:1n-7	0.0132 ± 0.0016	0.0088 ± 0.0006	0.0091 ± 0.0039			
18:1n-7	0.0914 ± 0.0100	0.0642 ± 0.0047	0.1045 ± 0.009	*		*
18:1n-9	0.0972 ± 0.0142	0.0935 ± 0.0048	0.0666 ± 0.0194			
20:1n-7	0.0015 ± 0.0004	0.0011 ± 0.0003	0.0006 ± 0.0002			
20:1n-9	0.0062 ± 0.0008	0.0045 ± 0.0006	0.0041 ± 0.0009			
20:1n-11	0.0029 ± 0.0009	0.0015± 0.0004	0.0011 ± 0.0004			
22:1n-7	0.0010 ± 0.0002	0.0010 ± 0.0006	0.0006 ± 0.0003			
22:1n-9	0.0029 ± 0.0007	0.0025 ± 0.0006	0.0018 ± 0.0004			
22:1n-11	0.0013 ± 0.0009	0.0006 ± 0.0003	0.0002 ± 0.0000			
24:1n-9	0.0011 ± 0.0004	0.0005 ± 0.0001	0.0008 ± 0.0003			
Total MUFA	0.2202 ± 0.02407	0.1798 ± 0.0100	0.1902 ± 0.0254			
18:2n-6	0.9149 ± 0.1041	0.5050 ± 0.0805	0.0851 ± 0.0215	**	****	**
18:3n-6	0.0005 ± 0.0002	0.0005 ± 0.0001	0.0002 ± 0.0000			
20:2n-6	0.0150 ± 0.0015	0.0107 ± 0.0015	0.0082 ± 0.0009	*	*	
20:3n-6	0.0199 ± 0.0032	0.0189 ± 0.0023	0.0071 ± 0.0029		*	*
20:4n-6	0.0125 ± 0.0019	0.0116 ± 0.0012	0.0082 ± 0.0010			
22:2n6	0.0007 ± 0.0004	0.0007 ± 0.0002	0.0006 ± 0.0002			
22:4n6	0.0013 ± 0.0004	0.0022 ± 0.0004	0.0004 ± 0.0000			**
22:5n-6	0.0037 ± 0.0013	0.0046 ± 0.0009	0.0008 ± 0.0002			
Total Omega-6	0.9686 ± 0.1098	0.5541 ± 0.0842	0.1045 ± 0.0234	**	****	**
18:3n-3	0.0062 ± 0.0007	0.0037 ± 0.0006	0.0040 ± 0.0009			
18:4n-3	0.0031 ± 0.0004	0.0012 ± 0.0004	0.0006 ± 0.0003	**	**	
20:3n-3	0.0004 ± 0.0001	0.0007 ± 0.0003	0.0005 ± 0.0001			
20:4n-3	0.0004 ± 0.0000	0.0005 ± 0.0001	0.0007 ± 0.0002			
20:5n-3	0.0012 ± 0.0003	0.0010 ± 0.0004	0.0008 ± 0.0003			
22:5n-3	0.0025 ± 0.0004	0.0031 ± 0.0010	0.0016 ± 0.0009			
22:6n-3	0.0331 ± 0.0084	0.0336 ± 0.0047	0.0125 ± 0.0019			
Total Omega-3	0.0469 ± 0.0096	0.0438 ± 0.0056	0.0201 ± 0.0036			
20:3n-9	0.0014 ± 0.0003	0.0006 ± 0.0002	0.0007 ± 0.0001			
Total CL	1.5090 ± 0.1414	1.0070 ± 0.1105	0.6705 ± 0.1064	*	**	

Data are means ± S.E.M; n = 4-5. Differences were analyzed by One-Way ANOVA; *P < 0.05, **P < 0.01, ****P < 0.0001.

Supplementary Table 2. Concentrations of cardiolipin fatty acyl species in heart tissue from female *Wt* and *Plaat1*$^{-/-}$ mice.

cardiolipin fatty acyl species	*Wt* (μg/mg of tissue)	*Plaat1*$^{-/-}$ (μg/mg of tissue)	*Wt* vs. *Plaat1*$^{-/-}$
10:0	0.0005 ± 0.0002	0.0006 ± 0.0003	
12:0	0.0033 ± 0.0009	0.0036 ± 0.0008	
14:0	0.0078 ± 0.0008	0.0067 ± 0.0008	
15:0	0.0097 ± 0.0031	0.0066 ± 0.0009	
16:0	0.1429 ± 0.0226	0.1165 ± 0.0062	
17:0	0.0062 ± 0.0006	0.0059 ± 0.0007	
18:0	0.0619 ± 0.0048	0.0664 ± 0.0096	
20:0	0.0018 ± 0.0006	0.0023 ± 0.0004	
22:0	0.0013 ± 0.0003	0.0020 ± 0.0006	
23:0	0.0009 ± 0.0001	0.0012 ± 0.0006	
24:0	0.0011 ± 0.0004	0.0017 ± 0.0004	
Total SFA	0.2378 ± 0.0283	0.2151 ± 0.0169	
12:1	0.0007 ± 0.0003	0.0008 ± 0.0002	
14:1	0.0014 ± 0.0003	0.0012 ± 0.0001	
16:1n-7	0.0139 ± 0.0013	0.0099 ± 0.0023	
18:1n-7	0.0932 ± 0.0030	0.0627 ± 0.0116	*
18:1n-9	0.0946 ± 0.0056	0.0811 ± 0.0103	
20:1n-7	0.0012 ± 0.0003	0.0009 ± 0.0004	
20:1n-9	0.0049 ± 0.0009	0.0036 ± 0.0008	
20:1n-11	0.0015 ± 0.0005	0.0007 ± 0.0002	
22:1n-7	0.0024 ± 0.0014	0.0017± 0.0005	
22:1n-9	0.0040 ± 0.0011	0.0034 ± 0.0009	
22:1n-11	0.0002 ± 0.0000	0.0003 ± 0.0001	
24:1n-9	0.0005 ± 0.0002	0.0016 ± 0.0006	
Total MUFA	0.2184 ± 0.0091	0.1667 ± 0.0240	
18:2n-6	1.0310 ± 0.0590	0.6051 ± 0.1027	*
18:3n-6	0.0005 ± 0.0002	0.0007 ± 0.0003	
20:2n-6	0.0162 ± 0.0014	0.0095 ± 0.0018	*
20:3n-6	0.0184 ± 0.0022	0.0111 ± 0.0022	
20:4n-6	0.0137 ± 0.0010	0.0112 ± 0.0024	
22:2n6	0.0008 ± 0.0003	0.0021 ± 0.0013	
22:4n6	0.0016 ± 0.0006	0.0021± 0.0012	
22:5n-6	0.0040 ± 0.0007	0.0022 ± 0.0013	
Total Omega-6	1.0860 ± 0.0622	0.6438 ± 0.1116	*
18:3n-3	0.0070 ± 0.0007	0.0046 ± 0.0006	*
18:4n-3	0.0028 ± 0.0008	0.0021 ± 0.0002	
20:3n-3	0.0013 ± 0.0004	0.0006 ± 0.0002	
20:4n-3	0.0010 ± 0.0004	0.0007 ± 0.0003	
20:5n-3	0.0012 ± 0.0006	0.0010 ± 0.0004	
22:5n-3	0.0020 ± 0.0007	0.0020 ± 0.0006	
22:6n-3	0.0388 ± 0.0029	0.0338 ± 0.0071	
Total Omega-3	0.0540 ± 0.0022	0.0438 ± 0.0082	
20:3n-9	0.0008 ± 0.0001	0.0004 ± 0.0001	*
Total CL	1.5970 ± 0.0915	1.070 ± 0.1345	*

Data are means ± S.E.M; n = 4. Differences were analyzed by One-Way ANOVA; *$P < 0.05$.

Supplementary Table 3. Primers used in cardiac gene expression analyses.

Gene	Forward (5'→3')	Reverse (5'→3')
β-actin	CAGCCACTGTCGAGT	TCCATGGCGAACTGGT
Gapdh	TGCACCACCAACTGCTTAGC	GGATGCAGGGATGATGTTCT
Col1a1	AAAACCACCAAGACCTCCCG	GAGGGAACCAGATTGGGGTG
Col3a1	ACGTAAGCACTGGTGGACAG	CAGGAGGGCCATAGCTGAAC
Fn	CTGCGCTCCATTCCACCTTA	GGTCGTACACCCAGCTTGAA
Il-1β	GCCACCTTTTGACAGTGATGA G	GACAGCCCAGGTCAAAGGTT
Il-6	GTCCTTCCTACCCCAATTTCCA	CGCACTAGGTTTGCCGAGTA
Tnfα	ATGGCCTCCCTCTCATCAGT	TTTGCTACGACGTGGGCTAC
IFNα	TGCCCAGCAGATCAAGAAGG	TCAGGGGAAATTCCTGCACC
IFNβ	GCACTGGGTGGAATGAGACT	AGGTACCTTTGCACCCTCCA
